# Surgery-Related Thrombosis Critically Affects the Brain Infarct Volume in Mice Following Transient Middle Cerebral Artery Occlusion

**DOI:** 10.1371/journal.pone.0075561

**Published:** 2013-09-24

**Authors:** Xiaojie Lin, Peng Miao, Jixian Wang, Falei Yuan, Yongjing Guan, Yaohui Tang, Xiaosong He, Yongting Wang, Guo-Yuan Yang

**Affiliations:** 1 Neuroscience and Neuroengineering Center, Med-X Research Institute and School of Biomedical Engineering, Shanghai Jiao Tong University, Shanghai, China; 2 Department of Radiology, Ruijin Hospital, School of Medicine, Shanghai Jiao Tong University, Shanghai, China; 3 Department of Neurology, Ruijin Hospital, School of Medicine, Shanghai Jiao Tong University, Shanghai, China; 4 School of Communication and Information Engineering, Shanghai University, Shanghai, China; University of Nebraska Medical Center, United States of America

## Abstract

Transient middle cerebral artery occlusion (tMCAO) model is widely used to mimic human focal ischemic stroke in order to study ischemia/reperfusion brain injury in rodents. In tMCAO model, intraluminal suture technique is widely used to achieve ischemia and reperfusion. However, variation of infarct volume in this model often requires large sample size, which hinders the progress of preclinical research. Our previous study demonstrated that infarct volume was related to the success of reperfusion although the reason remained unclear. The aim of present study is to explore the relationship between focal thrombus formation and model reproducibility with respect to infarct volume. We hypothesize that suture-induced thrombosis causes infarct volume variability due to insufficient reperfusion after suture withdrawal. Seventy-two adult male CD-1 mice underwent 90 minutes of tMCAO with or without intraperitoneal administration of heparin. Dynamic synchrotron radiation microangiography (SRA) and laser speckle contrast imaging (LSCI) were performed before and after tMCAO to observe the cerebral vascular morphology and to measure the cerebral blood flow *in vivo*. Infarct volume and neurological score were examined to evaluate severity of ischemic brain injury. We found that the rate of successful reperfusion was much higher in heparin-treated mice compared to that in heparin-free mice according to the result of SRA and LSCI at 1 and 3 hours after suture withdrawal (*p*<0.05). Pathological features and SRA revealed that thrombus formed in the internal carotid artery, middle cerebral artery or anterior cerebral artery, which blocked reperfusion following tMCAO. LSCI showed that cortical collateral circulation could be disturbed by thrombi. Our results demonstrated that suture-induced thrombosis was a critical element, which affects the success of reperfusion. Appropriate heparin management provides a useful approach for improving reproducibility of reperfusion model in mice.

## Introduction

A reproducible cerebral ischemia/reperfusion model is critical for the development of ischemic therapy in rodent. Early reperfusion is an indispensable prerequisite for the ischemic tissue recovery and animal survival. However, sudden tissue hyper-perfusion after ischemia is deleterious, leading to accelerated and additional tissue injury, which is called “reperfusion injury” [1-4]. It is important to develop a reproducible cerebral ischemia/reperfusion model in rodent for better studying the mechanisms of ischemic stroke and developing feasible therapies. Post-ischemic reperfusion occurs frequently in human ischemic stroke when occlusion was caused by embolism. To mimic this clinical situation, intraluminal suture transient middle cerebral artery occlusion (tMCAO) model was developed in rats and mice [5,6]. This tMCAO model, being widely accepted for reperfusion study, allows reperfusion after suture withdrawal.

Despite the advantages of suture tMCAO model, the reproducibility of this model remains unsatisfactory. There are several complications associated with this model, including inadequate occlusion of the MCA [7,8], subarachnoid hemorrhage (SAH) [8,9], and the high variation of posterior communicating arteries (PcomAs) [10,11]. In addition, the diameter of silicon-coated suture must be a bit greater than the inner diameter of vessel to prevent any flow around the suture [7,8]. Suture insertion or removal from ICA could cause endothelial cell perturbation and vessel wall disruption, which also affect the reproducibility of the suture MCAO model [9,12,13].

Collateral artery opening after ischemic stroke could attenuate neuronal damage and death both in clinical and pre-clinical research [14]. Suture MCAO model was widely used for studying the changes of collateral circulation. Wang et al found that some collateral arteries could open for 90 to 150 minutes after permanent MCAO, and some collateral arteries could permanently open in response to ischemia [15]. However, how long would collateral circulation open after reperfusion and whether failed reperfusion influences the opening of collateral circulation is largely unknown.

Synchrotron radiation microangiography (SRA) provides a unique tool for monitoring hemodynamic changes and micro-vascular morphology [16]. Recent developments in SRA showed that it could be utilized for high-resolution imaging of cerebral vasculature [11,17,18]. Laser speckle contrast imaging (LSCI) has been used to monitor blood flow and cortical collateral arteries after ischemic stroke [15,19]. In this study, we use these unique high-resolution imaging techniques to investigate whether suture insertion causes thrombus formation, influences the success of reperfusion and collateral circulation during reperfusion period in tMCAO model.

## Materials and Methods

### Surgical procedures

Animal surgical procedures and experimental protocols were reviewed and approved by the Institutional Animal Care and Use Committee (IACUC) and The Bioethics Committee of School of Biomedical Engineering, Shanghai Jiao Tong University, Shanghai, China. Seventy-two adult male CD-1 mice weighing 30-35 grams (Sippr-BK, Shanghai, China) were used in the study. Mice were randomly divided into heparin-treated group and saline-treated group. The surgical procedure of tMCAO was described previously [20]. Briefly, mice were anesthetized with Ketamine (100 mg/kg) and Xylazine (10 mg/kg) intraperitoneally. Mice were placed supinely on a heating pad (RWD Life Science, Shenzhen, China), which maintains body temperature at 37.0 ± 0.5°C. The left common carotid artery (CCA), the external carotid artery (ECA) and the internal carotid artery (ICA) were isolated. 6-0 suture (Dermalon, 1741-11, Covidien, OH) coated with silicone was introduced into the ECA stump and advanced from the ICA to the opening of MCA until a slight resistance was felt. At this moment, the tip of the suture was located in the anterior cerebral artery (ACA). All procedures were performed under an operating microscope (Leica, Wetzlar, Germany). The success of occlusion was characterized as the reduction of cerebral blood flow (CBF) down to 10% of baseline [11,21], which was verified by a laser Doppler flowmetry (Moor Instruments, Devon, England). Mice were injected with 0.1 ml (200 U/ml) heparin or saline intraperitoneally at 80 minutes after occlusion. The suture was removed 10 minutes after heparin injection.

### Synchrotron radiation microangiography (SRA)

SRA was conducted at the X-ray imaging beam line BL13W in Shanghai Synchrotron Radiation Facility (SSRF). SRA imaging parameters were described previously [11]. The X-ray (33.2 keV) for imaging has a transversal coherence length about 31 µm and a field of view up to 45 mm (Height) ×4.5 mm (Width). PCO X-ray CCD camera (pixel size 13 µm × 13 µm, PCO-TECH Inc., Germany) was used to record the image sequences during the experiment.

Mice were placed vertical to the beam path. Contrast agent Ipamiro (80 µl, 175 mgl/ml, Ipamiro, Shanghai, China) was injected into the CCA at a speed of 33.3 µl/second. Dynamic images were obtained at a speed of 7 images per second. Baseline images taken before the injection of contrast agent were used to perform flat-field correction of raw images taken after the injection of contrast agent by MatLab software (Mathworks Ltd., Natick, Mass). Each layer of image was finally stitched together to generate a full-field angiographic image by Photoshop software (Adobe, San Jose, CA).

To examine sub-cortical vascular morphology and the Circus Willis, mice were imaged using SRA before MCAO and 20 minutes after MCAO, and at 0, 1 or 3 hours after suture withdrawal.

### Laser speckle contrast imaging (LSCI)

LSCI was performed using a high resolution Laser Speckle Contrast Imaging System (LSCI-2 system, Dolphin BioTech Ltd., Shanghai, China). The imaging procedure was described previously [22]. After anesthetizing animal, a midline incision was made on the scalp, and pericranium was removed to expose the surface of the skull. The raw speckle images (696 × 512 pixels, 40 μm/pixel) were acquired at 23 fps (exposure time T= 5 ms) under 780 nm laser illumination. In each trial, 200 consecutive frames of speckle images were recorded. Image processing was carried out off-line using MatLab software. To reduce noise, we first aligned the raw LSCI images by the registered laser speckle contrast analysis (rLASCA) method [23]. Then registered speckle images were processed by the random process estimator method [22] to obtain the contrast image with improved Signal to Noise Ratio (SNR). The relative blood flow speed *v* was calculated from contrast values according to the theory of LSCI [24]. Finally, normalized blood flow image was obtained using Eq. 1. 

$\bar{V}=\frac{V}{V_{\max}}\times 100\%$ Eq. 1

*v* is the relative blood flow speed, *v*_*max*_ is the maximum value in the image of relative blood flow speed, $\bar{V}$ is the normalized blood flow speed.

Mice were imaged using LSCI for collateral circulation detection before and 10, 30 minutes after suture insertion and 10, 60 and 120 minutes after suture withdrawal both in heparin-treated and heparin-free mice. SRA and LSCI were performed on separate mice.

### Histological staining

**Figure 1:**
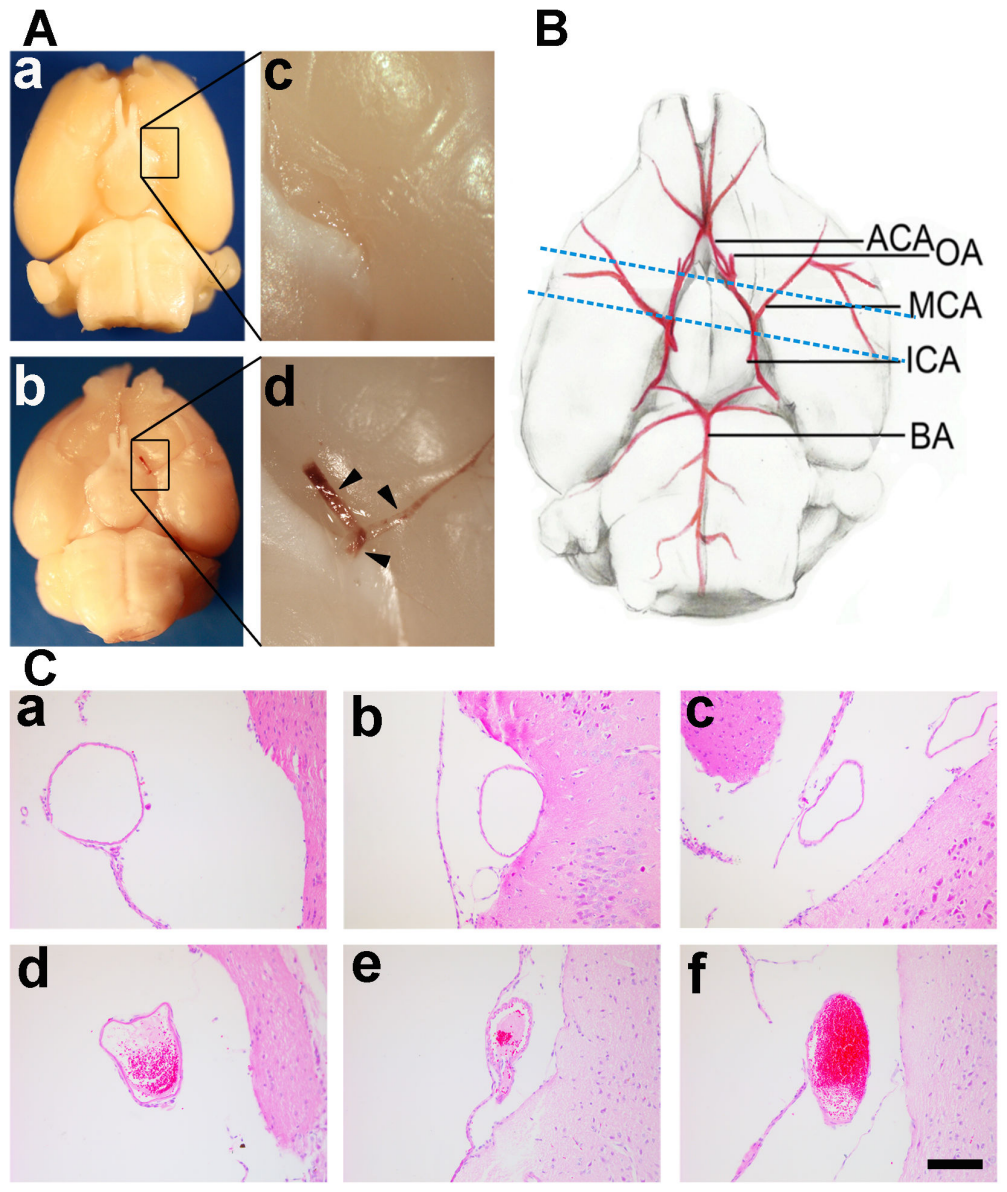
Thrombosis in vascular after reperfusion detected by HE-staining. (**A**) Stereomicroscopic imaging of brain was performed after 1 to 3 hours after reperfusion. No blood clotting was detected in the Circus Willis in successfully reperfused mice (**a**, **c**) while blood clotting was clearly detected in MCA, ACA and ICA territory in heparin-free mice with failed reperfusion (**b**, **d**). Arrowheads indicate thrombus in the MCA, ACA and ICA. (**B**) Schematic diagram of paraffin section indicating the location of ACA, MCA and ICA. (**C**) HE-staining shows the cross-sections of ICA (**a**), MCA (**b**) and ACA (**c**) in successfully reperfused mice with no thrombus in the lumen after reperfusion. In contrast, the cross-sections of ICA (**d**), MCA (**e**) and ACA (**f**) in mice with failed reperfusion have mixed thrombus in the lumen after suture withdrawal. Bar= 100 µm. ACA: anterior cerebral artery, ICA: internal carotid artery, MCA: middle cerebral artery.

After LSCI and SRA measurement, mice were anesthetized, then perfused with normal saline followed by 4% paraformaldehyde transcardially. The brain was removed and immersed in 4% paraformaldehyde for another 24 hours. Whole brain photographs were taken using a microscope (Leica, Wetzlar, Germany) to visually examine the existence of thrombus in cerebral arteries. Then the brain was dehydrated through a graded alcohol system and then embedded in paraffin for pathologic study. Paraffin sections were prepared for further histological staining. The schematic of section was shown in Figure **1B** as described previously [25]. Hematoxylin-eosin (HE) staining was used to detect the existence of thrombus.

### Neurological score

After 24 hours of tMCAO, neurological score was examined blindly. Neurological score was graded on a scale of 0 to 14 (normal score 0; maximal deficit score 14) [26]. One score point was assigned for the inability to perform the test or for the lack of a tested reflex.

### Triphenyltetrazolium chloride (TTC) staining

Mice were sacrificed following an overdose of ketamine injection after examination of neurological score. Brains were removed and sliced into 2 mm cross sections using a mouse brain matrix (RWD Life Science, Shenzhen, China). Brain sections were incubated in 2% TTC solution (Sigma, Santa Clara, CA) at 37 °C for 20 minutes. Brain slices were photographed, and infarct volume was calculated by summarizing contralateral hemisphere minus normal area of ipsilateral hemisphere times thickness as described previously [6].

### Statistical analysis

Data were presented as mean ± SD. Infarct volume between groups was compared by Student’s unpaired *t*-test. The rate of successful reperfusion was compared by chi-square test. A probability value of less than 5% was considered to represent statistical significance.

## Results

### SRA and LSCI measurements in mice following tMCAO

**Figure 2:**
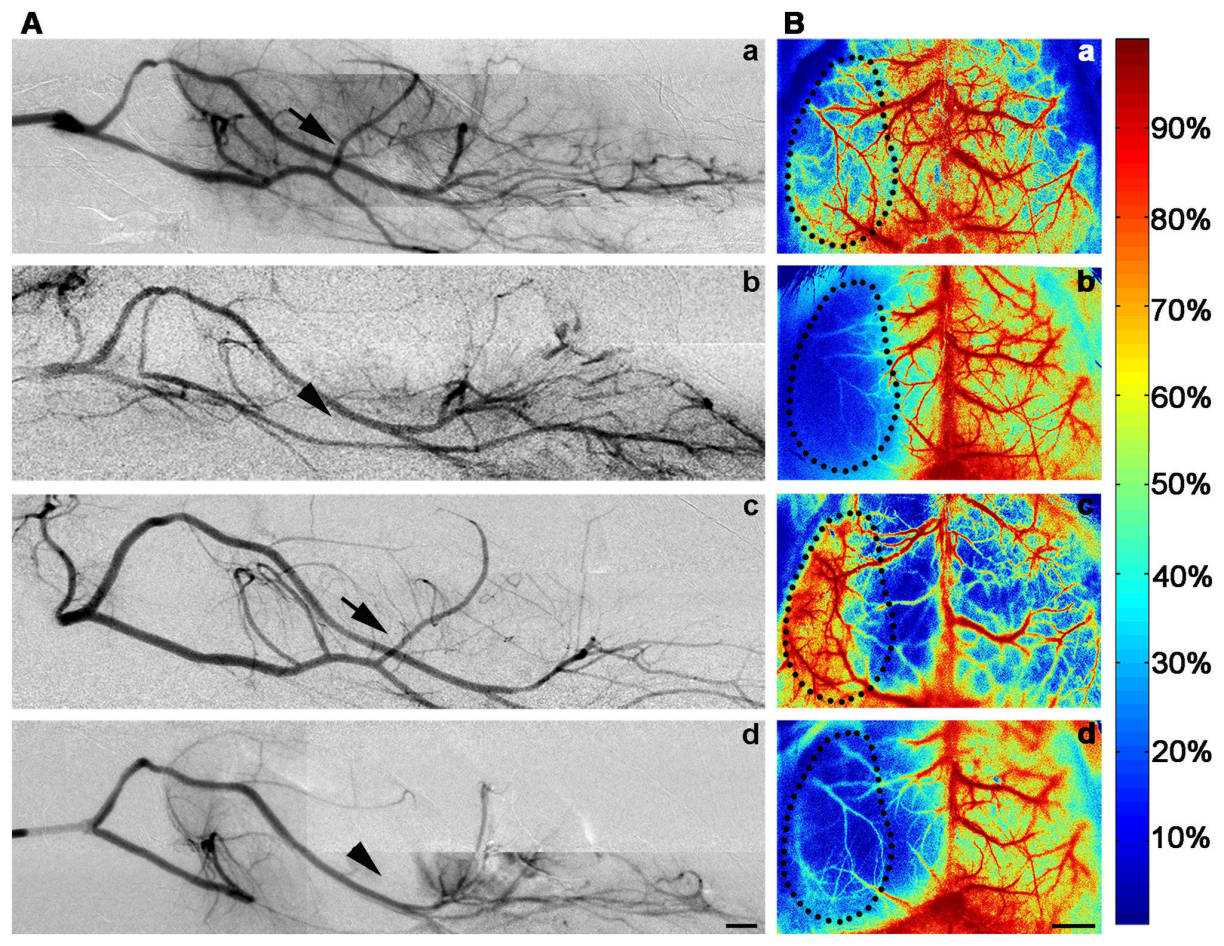
Mouse cerebral vascular morphology imaged by SRA and blood flow measured by LSCI. (**A**) Cerebral vascular morphology imaged by SRA. (**a**) cerebral vascular morphology was obtained before middle cerebral artery occlusion (MCAO) in adult mice. CCA, ICA, PPA, PCA, MCA and ACA as well as their branches could be clearly detected. (**b**) SRA after MCAO showed that CCA, ICA, PPA and PCA could be detected while MCA was missing, suggesting that MCA was occluded. (**c**) SRA was performed 1 hour after reperfusion in heparin-treated mice, which showed CCA, ICA, PPA, PCA and MCA were well filled with contrast agent Ipamiro. (**d**) SRA was performed 1 hour after reperfusion in the heparin-free mice, which showed CCA, ICA, PPA and PCA could be detected while MCA was still undetectable. (**B**) CBF was measured by LSCI. (**a**) LSCI was recorded in normal mice showing that the CBF of ipsilateral hemisphere was normal, similar to that of the contralateral. (**b**) LSCI was recorded after MCAO showing that the CBF of the ipsilateral hemisphere decreased to 10% of the baseline. (**c**) LSCI was recorded 1 hour after reperfusion in heparin-treated mice showing that the CBF recovered to the baseline. (**d**) LSCI recorded 1 hour after reperfusion in the heparin-free mice showing that the CBF was still low. Arrows indicate that MCA was well filled with blood. Arrowheads indicate the location of MCA, which was undetectable. Broken Circle indicated the MCA territory in the cortex of ipsilateral hemisphere. Bar= 1 mm. Color bar: normalized relative CBF speed (0%~100%). ACA: anterior cerebral artery, CBF: cerebral blood flow, CCA: common carotid artery, ICA: internal carotid artery, LSCI: laser speckle contrast imaging, PCA: posterior cerebral artery, PPA: pterygopalatine artery, SRA: synchrotron radiation microangiography.

Clear cerebral vascular morphology was observed, including ACA, MCA, posterior cerebral artery (PCA), pterygopalatine artery (PPA) and ICA in the mouse brain before tMCAO with MCA and ACA un-perfused after MCAO (Figure **2A**). MCA and ACA were detectable in heparin-treated mice within 60 minutes of reperfusion (Figure **2A, D**). In contrast, MCA and ACA could not be detected in the heparin-free mice after reperfusion.

To examine collateral blood supply, we measured CBF using LSCI following tMCAO. Normal CBF was observed in both hemispheres of the brain before MCAO while CBF in the ipsilateral MCA territory rapidly decreased after MCAO (Figure **2B**). It was observed that CBF in the ipsilateral MCA territory was even lower at 60 minutes after reperfusion than at the point of occlusion in the heparin-free mice while CBF was recovered at 60 minutes after reperfusion in heparin-treated mice (Figure **2B**).

### Endovascular thrombus was detected in mice with failed reperfusion

We performed HE staining to detect thrombus formation in the ACA, MCA, or ICA following reperfusion. We demonstrated that thrombosis occurred at multiple sites in mice with failed reperfusion in the heparin-free group while no thrombus was observed in the heparin-treated mice (Figure **1A**). Various degrees of mixed thrombus were observed in ICA, MCA and ACA in the heparin-free group (Figure **1C**). Vasospasm was not observed during SRA procedure or in histology results, suggesting that the failed reperfusion was mainly due to thrombosis but not transient elements such as contrast agent induced vasospasm.

### Thrombosis was the key effecter of reperfusion and reproducibility of tMCAO model

**Figure 3:**
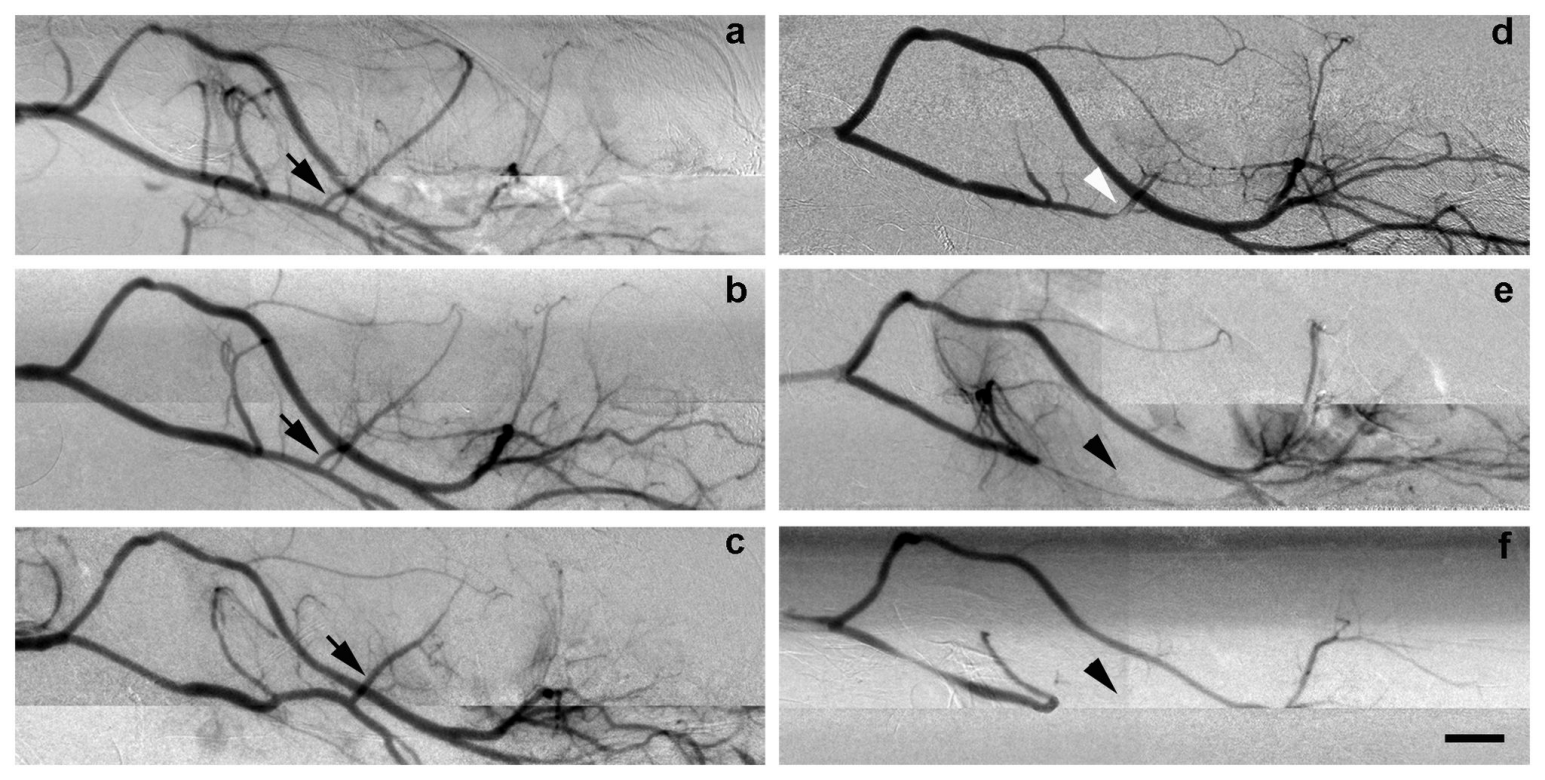
Mouse cerebral vascular morphology after tMCAO and at various time after reperfusion detected by SRA. SRA after 90 minutes of MCAO and at 0 (**a**), 1 (**b**) and 3 hours (**c**) after reperfusion in heparin-treated mice shows that the CCA, ICA, PPA, PCA, MCA and ACA could be clearly detected. In contrast, SRA after 90 minutes of MCAO and at 0 (**d**), 1 (**e**) and 3 hours (**f**) after suture withdrawal in heparin-free mice shows that the CCA, ICA, PPA and PCA could be clearly detected while the MCA and ACA could only be partially detected (**d**) or undetected (**e**, **f**). Arrows indicate that MCA was well filled with contrast agent Ipamiro. Black arrowheads indicate the location of MCA, which was undetectable. White arrowhead indicate the location of MCA, which was partially detected. Bar= 1 mm. ACA: anterior cerebral artery, CCA: common carotid artery, ICA: internal carotid artery, MCAO: middle cerebral artery occlusion, PPA: pterygopalatine artery, PCA: posterior cerebral artery, SRA: synchrotron radiation microangiography.

SRA and LSCI results further demonstrated that 13 mice failed reperfusion in the heparin-free group after 0, 1 or 3 hours of reperfusion (Figure **3**). MCA in two heparin-free mice was partially occluded immediately after reperfusion. In contrast, successful reperfusion was found in the heparin-treated mice at the same time of reperfusion. This result inferred that suture-induced thrombus formation could be a critical element leading to failed reperfusion after tMCAO.

SRA and LSCI experiments showed that the success rate of reperfusion was 50% to 75% within 3 hours of reperfusion in the heparin-free mice (Table **1**). In contrast, the success rate of reperfusion was 100% at each time point of reperfusion in heparin-treated mice. The success rate of reperfusion was significantly different between the heparin-treated and heparin-free mice at 1 or 3 hours after reperfusion (*p*< 0.05). There was no significant difference in CBF measurement between the two groups immediately after reperfusion (*p*>0.05), suggesting that thrombus gradually grew over time.

**Table 1:** The success rate of reperfusion after 90 minutes of MCAO and at 0, 1 and 3 hours after suture withdrawal in heparin-treated or heparin-free mice.

Group	0 hour	1 hour	3 hours
Heparin	100% (n=6)	100% (n=6)	100% (n=6)
Saline	75% (n=16)	68.8% (n=16)	50% (n=8)
*P* value	0.089	0.049*	0.016*

* *p*<0.05, representing significant difference between heparin-treated group and heparin-free saline group.

### Thrombosis affected cortical collateral circulation after reperfusion

**Figure 4:**
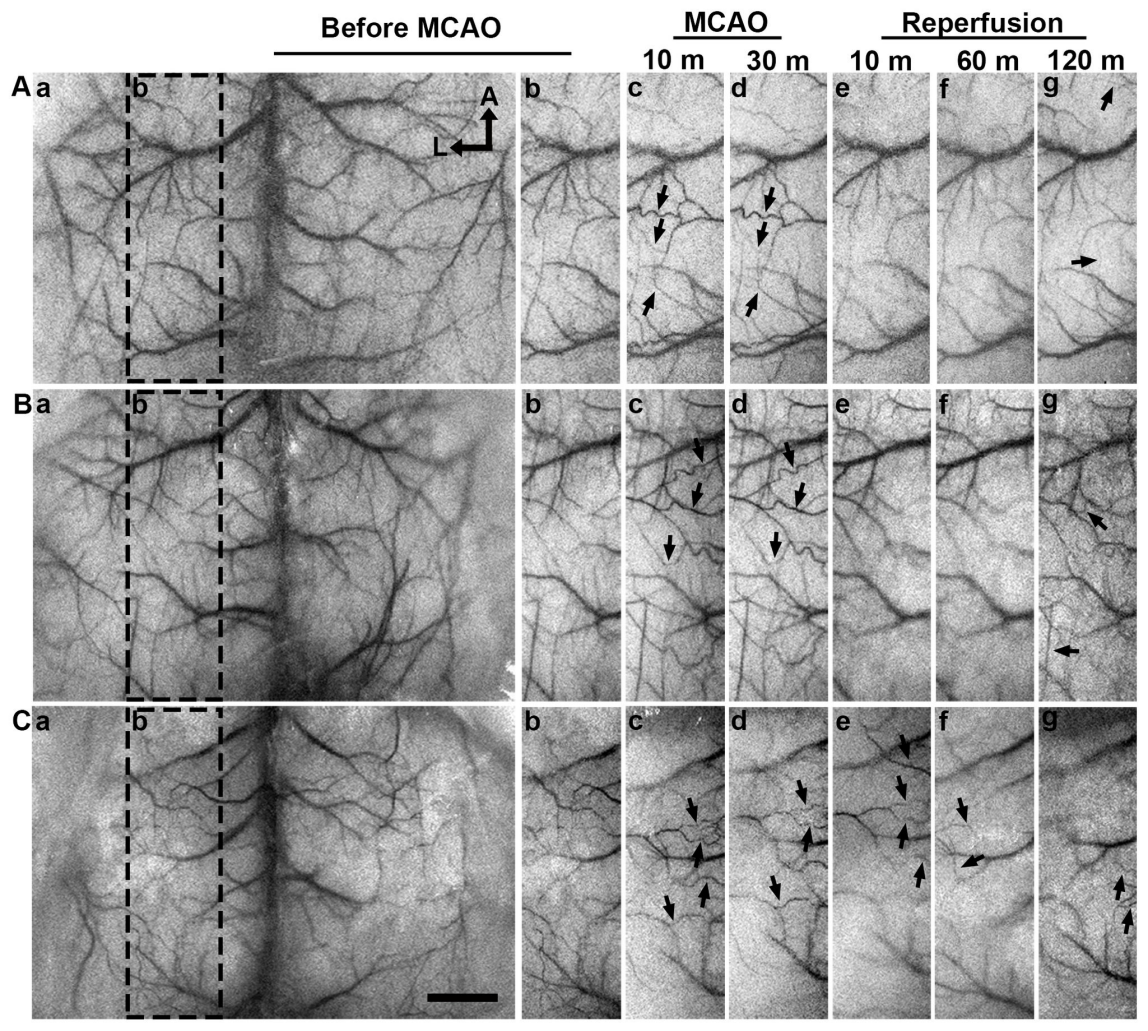
Cortical collateral circulation after tMCAO detected by LSCI. Cortical collateral circulation opened after tMCAO in successfully reperfused heparin-free mice (**A**) and heparin-treated mice (**B**). LSCI before MCAO (**a**-**b**) showed a clear cortical vascular morphology. Collateral arteries opened at 10 (**c**) and 30 minutes (**d**) after reperfusion. Collateral arteries were missing at 10 (**e**) and 60 minutes (**f**) after reperfusion. Notably, collateral arteries opened again at 120 minutes after reperfusion (**g**). (**C**) Collateral circulation after tMCAO in heparin-free mice with failed reperfusion. LSCI before MCAO (**a**-**b**) showed a clear cortical vascular morphology. Collateral arteries opened at 10 (**c**) and 30 minutes (**d**) after suture withdrawal. Collateral arteries were still open at 10 (**e**), 60 (**f**) and 120 minutes (**g**) after suture withdrawal. Broken Circle indicates the selected areas of images **b** to **g**. Arrows indicate the collateral artery. Bar= 1 mm. A: anterior, L: left, LSCI: laser speckle contrast imaging, tMCAO: transient middle cerebral artery occlusion.

LSCI was used to follow the time course of the opening of collateral arteries following MCAO and after suture withdrawal. LSCI showed that collateral arteries opened after MCAO (Figure **4**). Collateral arteries were no longer observed after 10 minutes of successful reperfusion (Figure **4B**). Some collateral arteries still open in the heparin-free mice after suture withdrawal due to failed reperfusion (Figure **4C**). It was noted that the collateral circulation opened again after 120 minutes of reperfusion both in mice with successful and failed reperfusion (Figure **4**).

### Thrombosis affected infarct volume and neurological score

**Figure 5:**
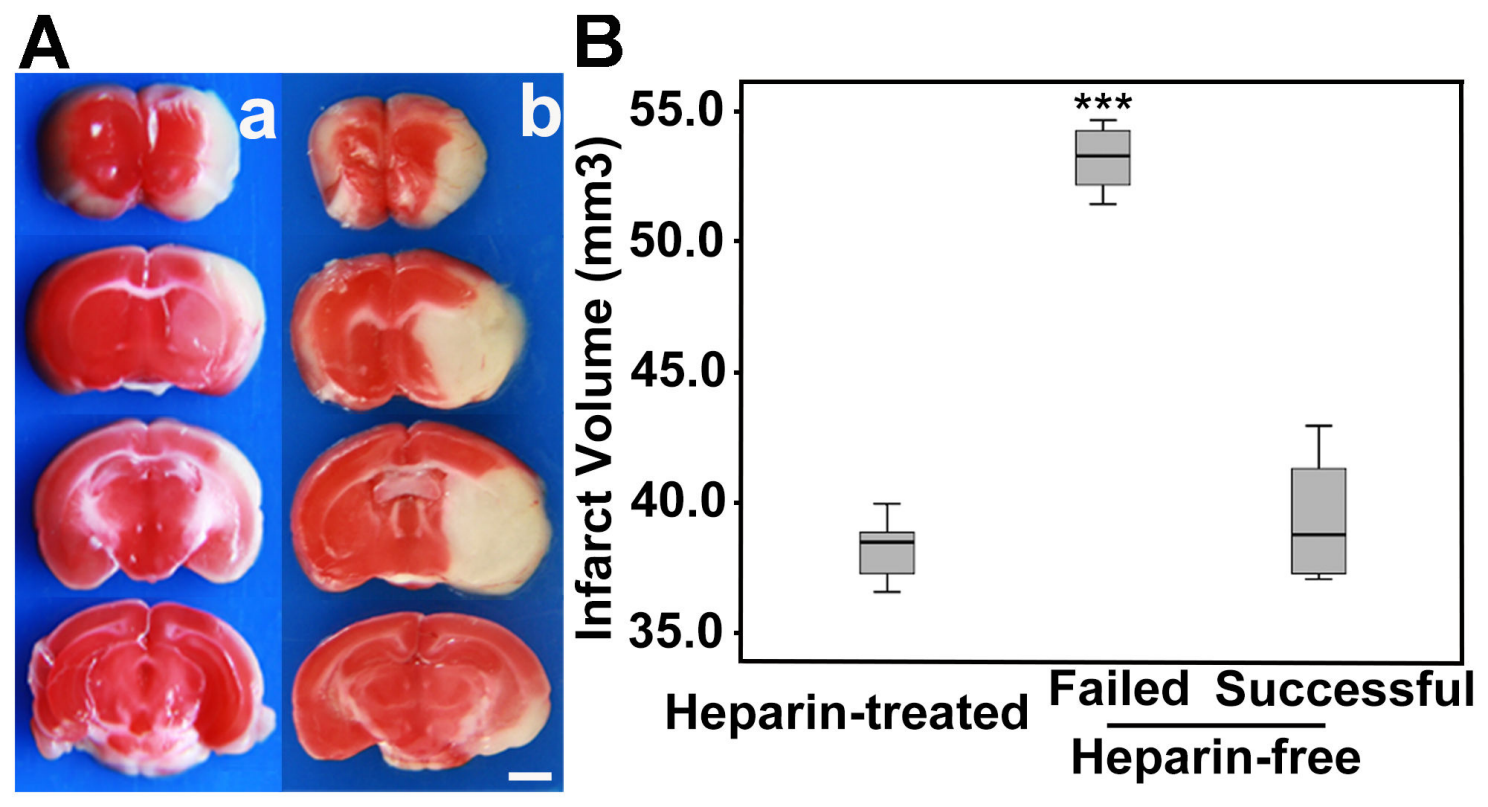
The infarct volume measured by TTC staining. (**A**) TTC staining showed the infarct territory 24 hours after reperfusion in successfully reperfused mice (**a**) and mice with failed reperfusion (**b**). (**B**) Quantitative data of the infarct volume in heparin-treated mice, heparin-free mice with failed reperfusion and successfully reperfused heparin-free mice, suggesting that the infarct volume in heparin-free mice with failed reperfusion (n=4) was significantly larger than heparin-treated (n=6) and successfully reperfused heparin-free mice (n=4). Data were mean±SD, ***p<0.001, Bar= 2 mm.

TTC staining and infarct volume measurements showed that the infarct volume was significantly larger in heparin-free mice with failed reperfusion than in successfully reperfused heparin-free mice and heparin-treated mice (Figure **5**, *p*<0.01). There was no significant difference in infarct volume between successfully reperfused heparin-free or heparin-treated mice (*p*>0.05). It was noted that the infarct volume was less variable in heparin-treated mice than in heparin-free mice.

**Figure 6:**
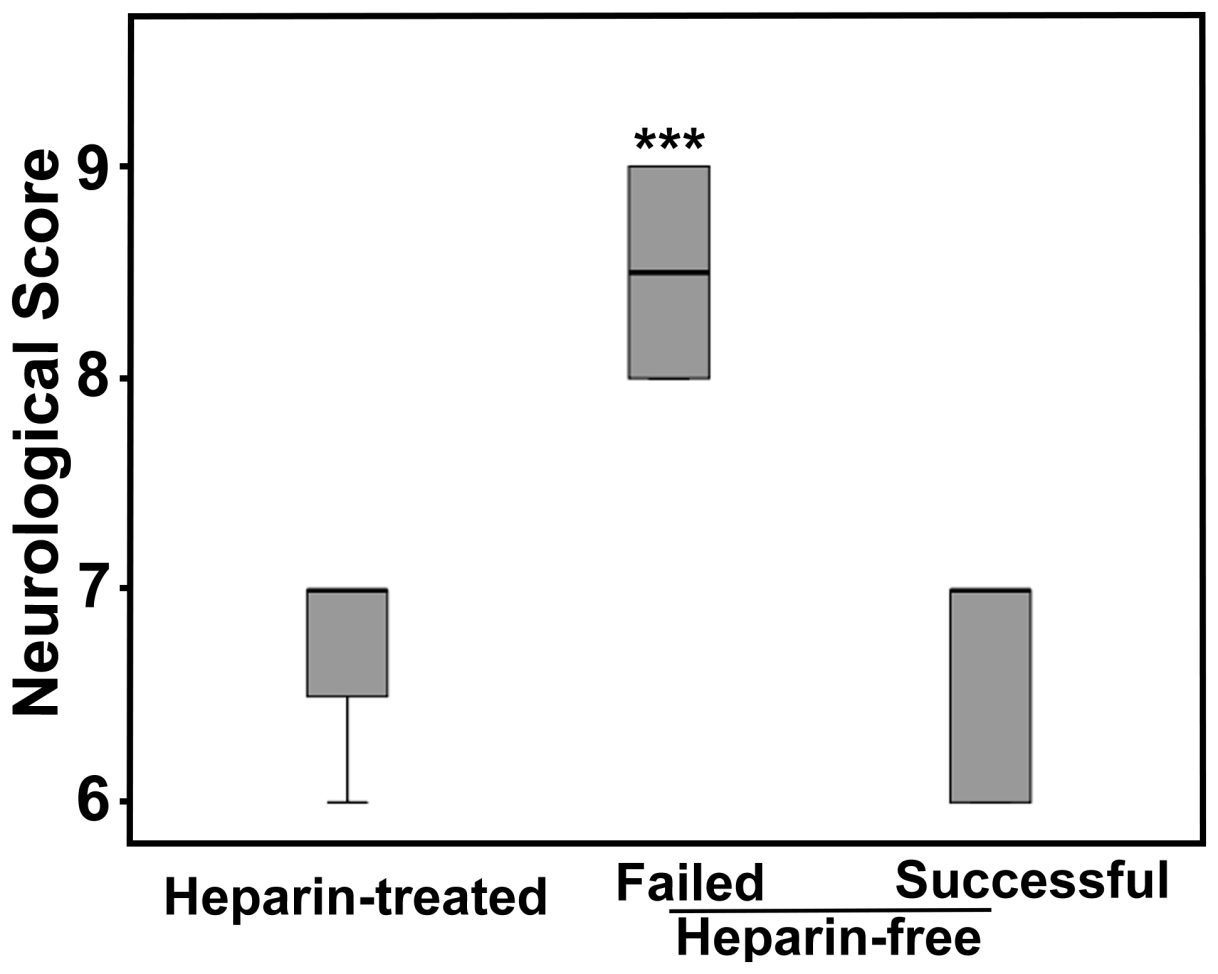
The neurological score at 24 hours after tMCAO. Quantitative data showed that the neurological score in heparin-free mice with failed reperfusion (n=4) was significantly higher than heparin-treated (n=6) and successfully reperfused heparin-free mice (n=4). Data were mean±SD, ***p<0.001.

Neurological score was significantly higher in heparin-free mice with failed reperfusion than in successfully reperfused heparin-free mice and heparin-treated mice (Figure **6**, *p*<0.01). There was no significant difference in neurological score between successfully reperfused heparin-free or heparin-treated mice. Heparin injection did not reduce the neurological score, but improved neurological score reproducibility after tMCAO.

## Discussion

Intraluminal suture induced tMCAO model is widely used in focal cerebral ischemic research. However, several model-inherent complications still exist: 1) filament insertion could result in inadequate MCAO, which was related to the shape and diameter of the suture [7,8]; 2) SAH could occur and complicate the pathophysiological relevance of the model [8,9]; 3) high mortality and infarct volume variability could occur in relation to the high variation of PcomAs [10,11]; and 4) hyperthermia during and post ischemia could complicate the interpretations of results [12,27].

Our results demonstrated that thrombus formation could affect the infarct volume and neurobehavioral outcomes, thus affect the reproducibility of transient suture MCAO model in rodents. All the mixed thrombus detected in this study was red thrombus, which might be explained by the perturbations of endothelia cells during the surgery started from the insertion of the suture to its withdrawal from ICA [9,12,13]. In this study, we provided direct evidence that reperfusion failed after suture withdrawal in some heparin-free mice, which was confirmed as thrombus occlusion by SRA and HE staining. We also found that the Circus Willis could be partially occluded in the heparin-free mice, which affected the model reproducibility. Vasospasm was not observed during dynamic imaging and histological staining, suggested that failed reperfusion was mainly due to newly formed thrombus. We found that the shape of MCA, ACA and ICA were changed after thrombus formation but not in successfully reperfused mice. It might be due to uneven dehydration of thrombi during paraffin section preparation.

Kawamura et al reported that thrombus formed in the ICA only when both the MCA and PPA were permanent occluded. They postulated that thrombus formation did not occur when the PPA was kept patent [28]. However, we found that thrombus occurred even when PPA was not ligated. This result suggested that the formation of thrombus was not limited to situations associated with PPA ligation.

Ischemia-induced collateral circulation could sustain brain tissue survival for hours after the occlusion of major arteries to the brain [14]. Therefore, more and more studies were carried out to understand collateral circulation after ischemic stroke both in clinical and pre-clinical setting [14,15,19,29,30]. Due to the variation of collateral circulation in rodents, model reproducibility and reliability was extremely important for developing new therapies to promote collateral flow [31]. In this study, we found that thrombosis could disrupt collateral circulation. Heparin injection before suture withdrawal successfully eliminated thrombus formation without disturbing the collateral circulation.

We used heparin injection to prevent thrombosis and demonstrated that the failure of reperfusion was due to thrombosis. However, heparin could significantly reduce infarct volume when injected before MCAO surgery [32]. In addition to thrombosis, inhibition of platelets after tMCAO may prevent endothelial cell dysfunction and brain-blood barrier damage [33,34]. We injected heparin 10 minutes before suture withdrawal to minimize the effects of heparin on infarct volume. TTC staining and neurological score test showed that such heparin treatment did not decrease brain infarct volume or neurological score. These results suggested that heparin injection could prevent thrombosis in ICA, MCA and ACA after suture withdrawal. Therefore, we propose that injecting heparin in tMCAO model could be used as an optional method in improving suture ischemia model.

We used flexible silicone suture because SAH occurs more often when rigid sutures were used [35]. Even slight differences in the diameter and quality of a suture could significantly affect lesion volume [9]. SAH was not detected in either heparin-treated or heparin-free group, suggesting that under our experimental conditions, treatment with heparin did not increase SAH incidence. Furthermore, incidence of animal death was zero.

Magnetic Resonance Imaging (MRI) is widely used to measure CBF and high-resolution Magnetic Resonance Angiography (MRA) is used to measure vessel patency in the entire process longitudinally [1,2,36-38]. However, the resolution of MRA is lower than SRA when used to detect vascular morphology, especially in rodents. LSCI is useful in evaluating cortical CBF during and after cerebral ischemia [39]. However, LSCI cannot observe vessels in the sub-cortex or the Circus Willis due to its limitation in detection depth. SRA can detect morphological changes of brain vasculature both in the cortex, the Circus Willis and the sub-cortex. SRA can be used to detect arteries with diameter less than 100 µm according to our previous study [40]. In contrast, other technologies including MRA cannot detect morphological changes in these vessels due to limitation in resolution [11,17,18,41]. Unfortunately, animals could not survive for extended period after SRA under current setting mainly due to radiation injury. We believe that SRA can be used to visualize the entire process of CBF longitudinally when radiation dose is preferably controlled in the future. We utilized both SRA and LSCI to detect CBF changes and vascular morphology including branches of MCA. We demonstrated that thrombus formation after suture withdrawal caused low reperfusion in the cortex, suggesting that thrombosis is a vital element that causes failure of reperfusion after tMCAO.
